# Diabetes classification model based on boosting algorithms

**DOI:** 10.1186/s12859-018-2090-9

**Published:** 2018-03-27

**Authors:** Peihua Chen, Chuandi Pan

**Affiliations:** 1 Institute of Biopharmaceutical Informatics and Technologies, Wenzhou Medical University, Wenzhou, China; 2 Department of Computer Technology and Information Management, The First Affiliated Hospital of Wenzhou Medical University, Wenzhou City, China

**Keywords:** Diabetes, Boosting algorithms, Computer-aided diagnoses

## Abstract

**Background:**

Diabetes mellitus is a common and complicated chronic lifelong disease. Hence, it is of high clinical significance to find the most relevant clinical indexes and to perform efficient computer-aided pre-diagnoses and diagnoses.

**Results:**

Non-parametric statistical testing is performed on hundreds of medical measurement index results between diabetic and non-diabetic populations. Two common boosting algorithms, Adaboost.M1 and LogitBoost, are selected to establish a machine model for diabetes diagnosis based on these clinical test data, involving a total of 35,669 individuals. The machine classification models built by these two algorithms have very good classification ability. Here, the LogitBoost classification model is slightly better than the Adaboost.M1 classification model. The overall accuracy of the LogitBoost classification model reached 95.30% when using 10-fold cross validation. The true positive, true negative, false positive, and false negative rates of the binary classification model were 0.921, 0.969, 0.031, and 0.079, respectively, and the area under the receiver operating characteristic curve reached 0.99.

**Conclusions:**

The boosting algorithms show excellent performance for the diabetes classification models based on clinical medical data. The coefficient matrix of the original data is a sparse matrix, because some of the test results were missing, including some that were directly related to disease diagnosis. Therefore, the model is robust and has a degree of pre-diagnosis function. In the process of selecting the preferred test items, the most statistically significant discriminating factors between the diabetic and general populations were obtained and can be used as reference risk factors for diabetes mellitus.

## Background

Diabetes mellitus is a metabolic disease that is caused by deficient insulin secretion or poor insulin utilization, with hyperglycaemia as the main symptom. The number of patients with type 2 diabetes has risen rapidly in recent years, especially in developing countries. Diabetes is a chronic, life-long disease that can be accompanied by many complications in its later stages. Most diabetic patients also have abnormal lipid metabolism, and hyperlipidaemia can easily lead to atherosclerosis, which can cause coronary heart disease, cerebral infarction and other serious complications of diabetes [1]. According to the latest data from the International Diabetes Federation, there are currently 4.15 hundred million adult diabetic patients worldwide, and the global cost for the treatment of diabetes and its complications reached $673 billion in 2015. Every 3 s, a person will be diagnosed as diabetic somewhere in the world, and every 7 s, a patient will die because of its complications. Furthermore, many people have abnormal glucose tolerance and without intervention and treatment will have developed diabetes after 5 years. Diabetes has become a public health problem worldwide [2, 3].

Data mining refers to the process of searching for and revealing information that has potential value from a large amount of data by using specific algorithms. At present, data mining technology has been widely used and applied in many fields of business and scientific research. With the continuous development of computer technology, medical treatment has gradually become digitized, and a large amount of medical data has been accurately recorded and preserved in medical institutions. These medical information resources are of great value for disease research [4, 5]. When using data mining technology to automatically and thoroughly analyse a large amount of historical data in a medical database, valuable medical diagnosis rules can be built [6]. Data mining technology, when applied to clinical medicine, can support the diagnosis, treatment, and prevention of disease [7–10]. Most algorithms in data mining can be used not only to classify or cluster diseases but also for feature selection to simplify the model and improve the computational efficiency during the modelling process [11, 12]. Some scholars use data mining algorithms to classify diabetes with clinical test data, such as HbA1c, adiponectin and BMI, and this approach achieves the desired results [13]. However, because of the complexity of the human body, the application of data mining technology in clinical medicine is still relatively limited in general.

A medical diagnosis analysis model built by data mining technology has the nature of artificial intelligence: it can exclude the interference of human factors, it has strong objectivity, and it can gradually standardize and automate the process of medical diagnosis [7, 14, 15]. The conclusion obtained by a clinical diagnosis model, which is established based on the clinical detection information of tens of thousands of patients over decades, can be more accurate than the conclusion drawn by an individual physician with limited cases in his career life. Hence, the application of data mining technology to clinical medical disease modelling facilitates disease diagnosis, providing relatively broad prospects for this application.

## Methods

### Data processing method and overall procedure

**Fig. 1:**
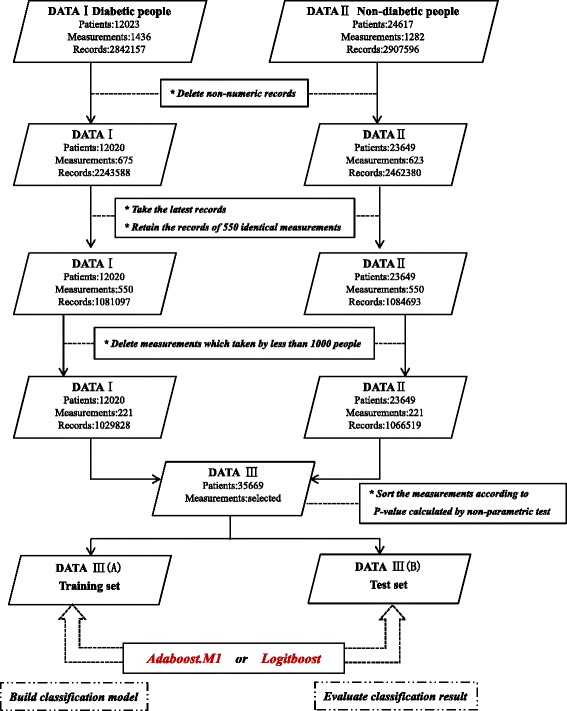
Data processing method and overall process

This study was a single-centre study, and the data were collected from the First Affiliated Hospital of Wenzhou Medical University. Clinical data were collected from more than 10,000 patients. These patients were diagnosed with diabetes in the endocrine department of the First Affiliated Hospital of the university and were hospitalized between July 2004 and April 2014. The physical examination data from more than 20,000 non-diabetic people were also collected. These individuals had a physical examination between October 2010 and August 2014. The collected clinical data set includes a large number of individuals and a wide range of physiological test indexes. The overall data processing flow is shown in Fig. 1.

The original collected data set contained two parts, DATA I, which contains the diagnosis and treatment data of the hospitalized diabetic patients, and DATA II, which comprises the physical examination data of the non-diabetic patients. DATA I contains the clinical test results of 12,023 patients, including 1436 medical test indexes and 2,842,157 test results. DATA II contains the clinical test results of 24,617 patients, including 1282 medical test indexes and 2,907,596 test results.

First, records were deleted in DATA I and DATA II if the recorded form was not in digital format. Second, if the patient had more than one result for the same test index, the latest record was used. The test records of 550 test indexes that were common to both DATA I and DATA II were then retained. Finally, of the 550 test indexes, the indexes where data were available for fewer than 1000 patients were removed. Afterward, DATA I and DATA II were combined to obtain the DATA III data set. For each of the test items, the clinical record was subjected to non-parametric statistical testing, and the Wilcoxon rank sum test was used. Using the DATA III data set, the Adaboost.M1 and LogitBoost algorithms were selected to build two category classifiers for the diabetes classification model; the performances were then tested. In the experiment, a 10-fold cross-validation method was employed.

### Two-category diabetes classification model based on Adaboost.M1 and LogitBoost

Machine learning has become a popular subject, and it can prejudge data by imitating the reasoning and thinking process of a human; thus, it helps people to make decisions. The goal of machine learning is to predict, as accurately as possible, unknown samples using a model or criterion that has been constructed from existing samples. Classification is a very common and important task in machine learning research. Traditional classification methods have many requirements with regard to the distribution of the raw data or existing information. For example, Fisher’s method requires that the data fit a multivariate normal distribution, and Bayes’ method requires that the probability distribution function and prior probability of each category are known. However, meeting these requirements is not always easy. Thus, when these conditions are not satisfied or the discriminant effect is poor, new methods are needed.

In 1990, Schapire proved that weak classifier performance, which is slightly better than random guessing, can be boosted to generate a strong classifier with arbitrary accuracy. Although building a high precision model is difficult, producing a classification model whose accuracy is only slightly better than random guessing (a “weak classifier”) is not difficult. The boosting algorithm is a machine learning method that uses pre-generated weak classifiers for continuous learning, gradually boosting them into a “strong classifier.”

The basic idea of the algorithm is to build a basic weak classifier, such as a decision tree, based on an existing sample data set; then, the weak classifier is called repeatedly, making the classifier more concerned with samples that are difficult to judge by giving greater weight to incorrectly judged samples in each round. After several rounds, synthetic strong classifiers are finally formed by giving weighted votes to the weak classifiers of each round, which provides a prediction model with high accuracy.

The boosting algorithms form a family of algorithms. Here, Adaboost and LogitBoost, two representative algorithms of this family, were selected to build the model. The Adaboost.M1 algorithm is a type of Adaboost algorithm where the value of the weak classifier is limited to {− 1,+ 1}, and the main process of the algorithm is shown below.

1. Input (*x*_1_, *y*_1_), (*x*_2_, *y*_2_), ⋯, (*x*_*n*_, *y*_*n*_)
2. Initialization $$ {W}_1=\left\{{W}_1(i)=\raisebox{1ex}{$1$}\!\left/ \!\raisebox{-1ex}{$n$}\right.,i=1,\cdots, n\right\} $$
3. For t = 1, 2, ⋯, T
  ① Get the weak learner: *h*_*t*_ : *X* → {−1, +1}
  ② Calculate the error of the classifier: $$ {E}_t=\raisebox{1ex}{$1$}\!\left/ \!\raisebox{-1ex}{$n$}\right.\sum {W}_t(i)I\left[{h}_t\left({x}_i\right)\ne {y}_i\right] $$
  ③ Calculate the weight of the classifier: $$ {a}_t=\raisebox{1ex}{$1$}\!\left/ \!\raisebox{-1ex}{$2$}\right.\mathit{\ln}\left[\left(1-{E}_t\right)/{E}_t\right] $$
  ④ Change the weight of the training samples: ${W}_{t+1}(i)={W}_t(i){e}^{-{a}_t{y}_i{h}_i\left({x}_i\right)}/{Z}_t$
4. Output $$ \mathrm{H}\left(\mathrm{x}\right)=\operatorname{sign}\left(\sum \limits_{t=1}^T{a}_t{h}_t(x)\right) $$

The training data is denoted by (*x*_1_, *y*_1_), (*x*_2_, *y*_2_), ⋯, (*x*_*n*_, *y*_*n*_) where *X*_*i*_ ∈ *R*^*d*^, *Y*_*i*_ ∈ {−1, +1}, *W*_*t*_(*i*) indicate the weight distribution of the samples at iteration t times. Furthermore, *Z*_*t*_ is the normalization factor, which ensures that the samples are subjected to one form of distribution.

The outstanding advantage of the Adaboost algorithm is its solid theoretical foundation, high prediction accuracy, and ease of implementation [16]. The LogitBoost algorithm is an improved form of the Adaboost algorithm; Adaboost uses an exponential loss function, while LogitBoost uses a negative log likelihood loss function. The basic idea of LogitBoost is similar to that of Adaboost, and as a result, we do not elaborate on it here.

In the classification model, we used the test indexes available for both the diabetic and non-diabetic patients as the features of the classification model, and we selected the detection results of the test indexes as the specific values of the features. If the patient did not participate in a test, then the test result was set to zero. The results for the two-category classification model were diabetic and non-diabetic. In the experiment, different groups of test items were taken as the features of the model, and the diabetes classification models were established using the above two types of boosting algorithm.

Logistic and RandomForest are very common data mining algorithms that can be used to classify clinical diseases. We select some parts of the clinical data (the clinical records of top 25 test index in 1000 diabetics and 1000 non-diabetics) tested by these two types of algorithms and the boosting algorithms used in this study to compare their classification effects.

The experimental results (Table 1) show that in clinical disease data modelling, the boosting algorithms used in this paper have advantages in terms of model accuracy and time consumption. In most cases, the accuracy of the Logistic and RandomForest algorithms are not as good as that of the boosting algorithms, and the modelling time is longer than that of the boosting algorithms. Among the selected algorithms, the Logistic algorithm had a much larger modelling time than the other selected algorithms.

**Table 1:** The classification effects of different algorithms

	Time taken to build model	The total computing time	Accuracy rate	ROC area
Logistic	23.31 S	43 M 22 S	85.35%	0.907
RandomForest	2.13 S	30 S	91.55%	0.978
AdaBoost.M1	0.07 S	1 S	92.6%	0.973
LogitBoost	0.33 S	1 S	93.93%	0.979

## Results

### Distribution of the values of important test indexes

In the research, a non-parametric test method, specifically, the Wilcoxon rank sum test, was used. The detection results for the common 221 test indexes for diabetics and non-diabetics were compared one-by-one to obtain a clinical medicine detection index that has significant differences between these two groups of people. Table 2 shows the top 30 most significant differences in the detection indexes among these groups.

**Table 2:** Top-30 clinical detection indexes of the most significant differences between diabetic and non-diabetic patients, and the *P* value of the non-parametric test

No.	Measurement name	*P*-value
1	Mean Corpuscular Hemoglobin Contentration	≈0
2	Packed Cell Volume (PCV)	≈0
3	Percentage of Lymph	≈0
4	Erythrocyte	≈0
5	Hemoglobin	≈0
6	Albumin	≈0
7	Total Protein	≈0
8	Albumin Globulin Ratio	≈0
9	Urea Nitrogen	≈0
10	Hdl-Cholesterol	≈0
11	Glucose	≈0
12	Electrical Conductivity	≈0
13	Tri-Iodo Thyronine	≈0
14	Calcium Serum	≈0
15	Free T3	≈0
16	Glycosylated Hemoglobin	≈0
17	Glucose (Emergency)	≈0
18	2 h Postprandial Blood Glucose	≈0
19	1 h Postprandial Blood Glucose	≈0
20	Erythrocyte Sedimentation Rate (ESR)	2.17E-300
21	Percentage Of Neutrophil	2.03E-290
22	Thrombin Time Ratio	9.91E-276
23	Indirect Bilirubin	2.46E-264
24	Gamma Glutamyltransferase	2.94E-249
25	Thrombin Time	9.46E-247
26	Fibrinogen	1.80E-241
27	Alkaline Absolute Value	1.82E-224
28	Cancer Embryo Antigen	1.11E-217
29	Absolute Value Of Lymph	2.87E-213
30	Serum Chloride	1.39E-203

The detection results of the 30 test items in the table were significantly different between the diabetic and non-diabetic populations (*P* < 0.01). Of these 30 test items, five indexes were directly related to the judgement of whether the person had or did not have diabetes. The specific indicators were indexes 11 (glucose), 16 (glycosylated haemoglobin, HbA1c), 17 (emergency glucose), 18 (2-h postprandial blood glucose), and 19 (1-h postprandial blood glucose). The other 25 indexes are currently rarely mentioned in clinical practice when diagnosing diabetes. However, the statistical results showed an enormous difference in the examination results of these 25 test indexes for the diabetic and non-diabetic populations. This finding indicates that in addition to the lower glucose uptake and utilization and significantly increased blood glucose and glycated haemoglobin, some other body functions and the corresponding indexes also exhibit large significant changes during or after the person develops the disease compared to those of the healthy population.

**Fig. 2:**
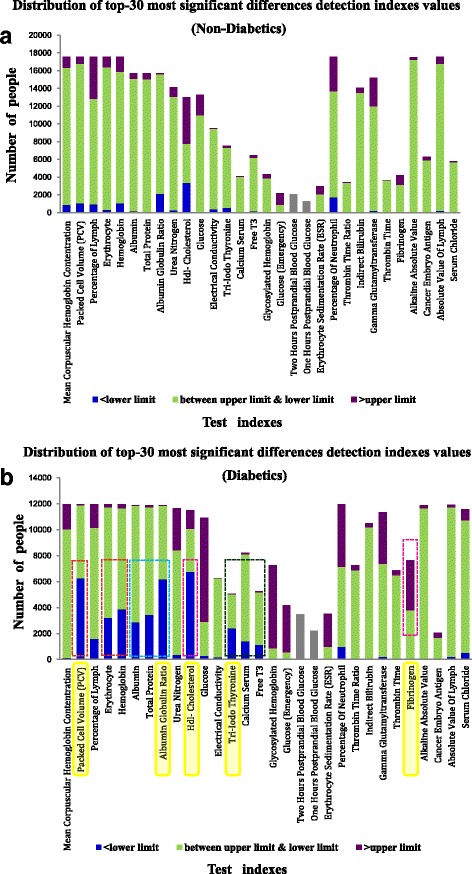
Examination results of important detection indexes of non-diabetic patients (**a**) and diabetic patients (**b**)

Five and 1806 people in the diabetic and non-diabetic populations, respectively, did not participate in the examination of any of the 30 extracted test items. All of the examination results of each test index were compared with the standard reference upper and lower limit values. Any examination result that was higher than the maximum value was labelled “>upper limit”; any result lower than the minimum value was labelled “<lower limit”, and any result between the maximum and minimum values was labelled “between upper limit & lower limit”. The final results are shown in Fig. 2.

Rough comparison of the test results of the 30 test indexes in the diabetic and non-diabetic populations in Fig. 2(a) and (b) indicated that for quite a number of tests, the proportion of diabetic patients who had results that were lower or higher than the normal value was higher than the corresponding proportion of non-diabetic individuals. In other words, an extremely large number of diabetic patients had abnormal test results for some test indexes.

Relative to the non-diabetic population, diabetic patients are prone to having high results for the following test indexes: urea nitrogen, glucose, HbA1c, glucose (emergency), erythrocyte sedimentation rate (ESR) and fibrinogen; these patients are also prone to having low results for the following test indexes: packed cell volume (PCV), erythrocyte, haemoglobin, albumin, total protein, albumin globulin ratio, HDL-cholesterol, triiodothyronine, serum calcium, and free T3.

Five test indexes are directly related to diabetes. Among them, the results of two test indexes, 1-h postprandial blood glucose and 2-h postprandial blood glucose, were not compared with the standard reference values because few patients had these results. For most of the diabetic patients, the examination results of the tests for glucose, HbA1c, and emergency glucose were higher than the normal upper limits. Three test indexes had the highest proportion of individuals whose examination results were higher than normal in the diabetic population, which is consistent with expectations.

In segments of the diabetic population, the glucose values were normal. For a small segment of the diabetic population, the glucose levels were lower than normal. The ability to control blood glucose in diabetic patients is not very good, and blood sugar values could be very different at different times of the day, such as in the early morning or evening, before or after a meal, and before or after taking medicine. The fluctuation in blood glucose is sometimes large. Under the action of insulin or hypoglycaemic drugs, blood sugar values can be kept within the normal range. However, in the case of an overdose of insulin or hypoglycaemic drugs, hypoglycaemia could also occur.

Some individuals in the non-diabetic population also had high blood glucose or HbA1c values. These people could be people with impaired glucose tolerance who are potentially diabetic. This situation could also occur because of a transient increase in blood glucose in a non-diabetic individual for some other reason.

For the PCV, albumin globulin ratio, and HDL-cholesterol test indexes, almost half or more than half of the patients with diabetes had low test results. For the triiodothyronine [17] and fibrinogen indexes [18], nearly half of the diabetic population had abnormal test results. The proportion of the non-diabetic population who had abnormal results for these test indexes was far smaller than the proportion of the diabetic population. Therefore, these indicators can be used as important reference risk factors for diabetes.

Further evaluation of the test indexes that had a high proportion of abnormal results showed that relative to that of non-diabetic patients, the body functions of diabetic patients experience some remarkable changes in the following respects. A) Anaemia, as indicated by the PCV, erythrocyte and haemoglobin are prone to be low [19, 20]. B) The nutritional status, as indicated by albumin, total protein, and the albumin globulin ratio, is prone to be poor. C) The risk of having cardiovascular disease is high, as shown by frequent low values of HDL-cholesterol and high values of fibrinogen [21, 22]. The results show that the complications that diabetic patients easily develop, such as hypertension, hyperlipoidaemia, coronary heart disease, and other vascular lesions, are most relevant to the reduced HDL-cholesterol rather than the increased LDL-cholesterol [23] and increased triglycerides [24, 25]. D) Hypothyroidism, or hypoparathyroidism, as shown by triiodothyronine and free T3, is a frequent finding. Furthermore, many diabetic patients have low serum calcium levels. A decrease in the function of the parathyroid gland decreases can lead to a decrease in serum calcium content.

### Performance of the classification model

The Data III data set contains 221 test indexes for both non-diabetic patients and diabetic patients. The test indexes were sorted in descending order according to the level of difference, i.e., according to the *P*-value calculated by the non-parametric statistical test. We used the top 100 test indexes with the most significant differences between the two populations to build the disease classification model.

**Fig. 3:**
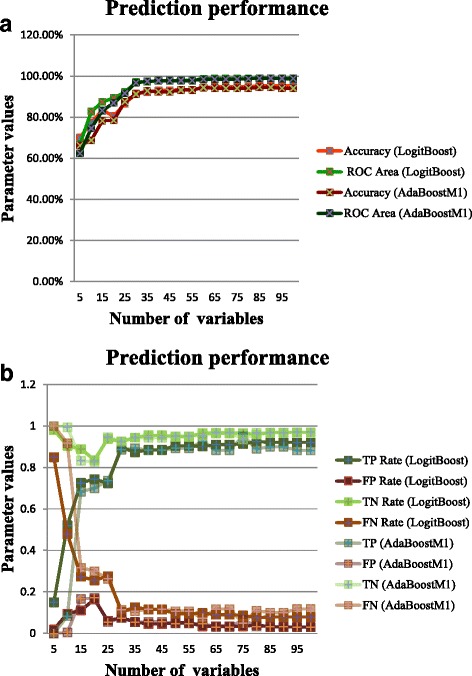
**a** Prediction accuracy and receiver operating characteristic (ROC) area of the models built by each boosting algorithm. **b** TP, FP, TN, and FN coefficients of the model built by each boosting algorithm

Figure 3(a) shows the final classification accuracy and receiver operating characteristic (ROC) area of the disease classification model built by the Adaboost.M1 and LogitBoost algorithms at each step, from 5 to 100 test indexes in increments of 5, using 10-fold cross validation. As the number of test indexes increased, the classification accuracy of the model rapidly rose. When the number of test indexes reached 30, the classification accuracy was greater than 90%, and the ROC area was greater than 97%. The models built by each boosting algorithm achieved very good classification results. However, the performance of the LogitBoost algorithm was slightly better.

Figure 3(b) shows the true positive (TP), true negative (TN), false positive (FP), and false negative (FN) rates at each step of the classification model built by the Adaboost.M1 and LogitBoost algorithms. With the use of at least 30 indicators, the models established by the two boosting algorithms have low rates of missed diagnosis and misdiagnosis.

The classification results show that using information from more test indexes can greatly improves the classification accuracy. In fact, when excluding the indexes that directly relate to the clinical diagnosis of the disease, the models built by the two boosting algorithms still have high classification accuracy, even when only the 25 test indexes of the top-30 indicators that are not directly related to the clinical diagnosis of the disease were used to build the classification model. When the Adaboost.M1 algorithm was used, the overall classification accuracy and ROC area were 89.10% and 0.958, respectively. When the LogitBoost algorithm was used, the overall classification accuracy and ROC area were 89.63% and 0.963, respectively.

These results show that the models can still provide a comparatively accurate judgement about an individual’s disease without the test results of the criteria indexes for that disease, such as glucose or HbA1c. Furthermore, some segments of the population did not participate in one or more examinations of the 25 tests. This finding also indicates that the models are robust and have a certain pre-diagnosis function. Furthermore, these results also illustrate that besides the glucose and glycated haemoglobin indexes, these 25 test indexes have a strong representational ability for diabetes.

## Discussion

The data evaluated in our study were obtained from thousands of patients, and hundreds of medical measurement indexes were involved. This research was a comprehensive study that involved a large amount of data. However, if the original data set was larger, for example, involving tens of thousands or hundreds of thousands of patients, and included almost all of their test results, the conclusions of the same analysis might be slightly different, i.e., some other detection indicators might also be present in the top 30 indexes.

In the classification model, the use of at least 30 detection indicators guaranteed the ideal classification accuracy. If all of the detection indicators are used, then more useful information can be provided, and the accuracy of the classification will be improved. There are five related indicators among the 30 indicators, and the other indicators were not considered as reference or related indicators when judging whether or not a person has diabetes in clinical practice. However, the statistical results show that as a healthy person gradually develops into a diabetic patient and as a person’s blood sugar is stable and continuously increasing, some body functions of the person and the corresponding test indexes also experience fundamental changes. The use of these test results could also provide an ideal classification result, i.e., diabetic patients could be identified, even in the absence of the person’s glucose and HbA1c data.

If more complete test results of a healthy population, population with impaired glucose tolerance, and diabetic population are used, then it could be possible to discover clinical indexes that are abnormal before the glucose or HbA1c indexes become abnormal, which would establish an early screening model for diabetes.

The Adaboost algorithm is one of the 10 most famous data mining algorithms, and it has been widely applied in areas such as face recognition [26]. The experimental results of this study show that this algorithm and LogitBoost, which is an improved version of Adaboost, show excellent performance in the modelling of disease classification by using clinical medical data.

## Conclusions

We found that the test indexes PCV, albumin globulin ratio, HDL-cholesterol, triiodothyronine, and fibrinogen differ significantly between people with and without diabetes; hence, these indexes can be used as important reference risk factors for diabetes mellitus.

Analysis and summary of the abnormal phenomena among the test indexes showed that patients with diabetes tend to be anaemic, have poor nutritional status, often suffer from cardiovascular disease, and tend to have decreased thyroid or parathyroid functions.

With the clinical test results of 35,669 patients, we used Adaboost.M1 and LogitBoost to build machine-classification models for diabetes. Both typical boosting algorithms showed excellent performance when used for disease classification modelling based on large amounts of clinical data.

## Abbreviations

ESR: Erythrocyte sedimentation rate
FN: False negative
FP: False positive
HbA1c: Glycosylated haemoglobin
PCV: Packed cell volume
ROC: Receiver operating characteristic
TN: True negative
TP: True positive
